# A Thermostable *Salmonella* Phage Endolysin, Lys68, with Broad Bactericidal Properties against Gram-Negative Pathogens in Presence of Weak Acids

**DOI:** 10.1371/journal.pone.0108376

**Published:** 2014-10-07

**Authors:** Hugo Oliveira, Viruthachalam Thiagarajan, Maarten Walmagh, Sanna Sillankorva, Rob Lavigne, Maria Teresa Neves-Petersen, Leon D. Kluskens, Joana Azeredo

**Affiliations:** 1 Centre of Biological Engineering, University of Minho, Braga, Portugal; 2 School of Chemistry, Bharathidasan University, Tiruchirappalli, India; 3 Laboratory of Gene Technology, Katholieke Universiteit Leuven, Leuven, Belgium; 4 Nanomedicine Department, International Iberian Nanotechnology Laboratory, Braga, Portugal; 5 Medical Faculty, Aalborg University, Aalborg, Denmark; University of the Basque Country, Spain

## Abstract

Resistance rates are increasing among several problematic Gram-negative pathogens, a fact that has encouraged the development of new antimicrobial agents. This paper characterizes a *Salmonella* phage endolysin (Lys68) and demonstrates its potential antimicrobial effectiveness when combined with organic acids towards Gram-negative pathogens. Biochemical characterization reveals that Lys68 is more active at pH 7.0, maintaining 76.7% of its activity when stored at 4°C for two months. Thermostability tests showed that Lys68 is only completely inactivated upon exposure to 100°C for 30 min, and circular dichroism analysis demonstrated the ability to refold into its original conformation upon thermal denaturation. It was shown that Lys68 is able to lyse a wide panel of Gram-negative bacteria (13 different species) in combination with the outer membrane permeabilizers EDTA, citric and malic acid. While the EDTA/Lys68 combination only inactivated *Pseudomonas* strains, the use of citric or malic acid broadened Lys68 antibacterial effect to other Gram-negative pathogens (lytic activity against 9 and 11 species, respectively). Particularly against *Salmonella* Typhimurium LT2, the combinatory effect of malic or citric acid with Lys68 led to approximately 3 to 5 log reductions in bacterial load/CFUs after 2 hours, respectively, and was also able to reduce stationary-phase cells and bacterial biofilms by approximately 1 log. The broad killing capacity of malic/citric acid-Lys68 is explained by the destabilization and major disruptions of the cell outer membrane integrity due to the acidity caused by the organic acids and a relatively high muralytic activity of Lys68 at low pH. Lys68 demonstrates good (thermo)stability properties that combined with different outer membrane permeabilizers, could become useful to combat Gram-negative pathogens in agricultural, food and medical industry.

## Introduction

Gram-negative bacterial pathogens are common causes of food-borne (e.g. *Salmonella*, *Escherichia coli* O157:H7, *Shigella*) and hospital-acquired (e.g. *Pseudomonas*, *Acinetobacter*) infectious diseases [1], [2]. In an era in which the threat of antibiotic and multi-resistant bacteria is increasing and solutions are becoming scarce, it is important to search for alternative antimicrobials.

One promising alternative approach to prevent or destroy pathogenic bacteria is the use of bacterial cell wall hydrolases. These enzymes cause bacteriolysis by degrading the peptidoglycan (PG) layer, also known as murein, the major component of the bacterial cell wall and responsible for the mechanical integrity. Among these bacterial cell wall hydrolases, an increased interest has been given to bacteriophage (phage) endolysins. Endolysins are specialized PG-degrading enzymes, part of a universal lytic cassette system encoded by all double stranded DNA phages and expressed during the terminal stage of the reproduction cycle [3].

In contrast to their extensively knowledge and successful use in fighting Gram-positive pathogens (e.g. *Streptococcus*, *Staphylococcus* and *Bacillus*) [4], the application of endolysins against Gram-negative pathogens has been impaired by the presence of a protecting outer membrane (OM), preventing their entry into the cell and reach the PG [5]. EDTA is a well-known outer membrane permeabilizer (OMP) that acts as a chelator by removing stabilizing cations from the OM, notably Ca^2+^ and Mg^2+^ [10]. EDTA (at 0.5 mM) is the only OMP agent used so far, to enhance the activity of these bacterial cell wall hydrolases through OM permeabilization, although with a moderate and narrow bacterial host effect limited to *P. aeruginosa* species [6], [7], [8], [9]. Organic acids are also reported to be membrane-active agents and hence potential permeabilizers [10]. Although some organic acids have, to a lesser extent, chelating properties, additional acidity can contribute to OM disruption [11]. Examples are citric acid (at 2 mM) and malic acid (at 5 mM) that were recently shown to weaken the OM of Gram-negative bacteria [12]. They are natural occurring compounds, versatile and widely used in food, cosmetic and pharmaceutical industries [10].

In this work it was intended to (*i*) biochemically characterize a new *Salmonella* phage endolysin (further abbreviated as Lys68), to enlarge the knowledge of a group of endolysins from Gram-negative affecting phages that remain scarcely explored and (*ii*) to develop an efficient anti-Gram-negative pathogen strategy by combining the endolysin with citric or malic acid and compared with EDTA, as it is the only OMP described so far to act synergistically with endolysins. We further present an explanatory hypothesis of the mechanism involved in the high and broad lytic ability of this endolysin in the presence of organic acids.

## Materials and Methods

### Bacteria, phage and chemicals

Bacterial strains used in this work are listed in **Table S1** and belong to the Centre of Biological Engineering bacterial collection (Braga, Portugal). All strains were grown in Lysogeny broth (LB) (Liofilchem, Italy) at 37°C and 120 rpm, with exception of *P. fluorescence* at 25°C. The *Salmonella* phage phi68 was isolated from faeces from a poultry farm (Braga, Portugal), and was recently characterized [13]. Hen egg white lysozyme (HEWL) (Fisher Scientific, USA), EDTA (Pronalab, Mexico) and HEPES, citric and malic acid (Sigma-Aldrich, USA) were purchased from the specified suppliers.

### Cloning, protein overexpression and purification

The isolated *Salmonella* phage phi68 was partially sequenced showing resemblance to the sequenced *Salmonella enterica* serovar Enteritidis typing phage SETP3 (data not shown). Based on phage SETP3 genomic sequence available at NCBI database (reference sequence: NC_009232.2), primers (Invitrogen) were designed to amplify the putative endolysin gene by PCR from the isolated phage phi68 genomic DNA template, using Phusion High-Fidelity DNA Polymerase (NEB, UK). Forward primer (AGATATCATATGTCAAACCGAAACATTAGC) and reverse primer (GTGGTGCTCGAGCTACTTAG) contained *Nde*I and *Xho*I restriction sites, respectively (underlined) [13]. The PCR amplification product was purified (DNA Clean & Concentrator-5k, Zymo Research, USA) and digested using *Nde*I and *Xho*I enzymes (NEB, UK), and cloned in the pET-28a expression vector (Novagen) with an N-terminal His_6_ tag. The presence of the insert in the plasmid was confirmed by DNA sequencing (Macrogen, Amsterdam) (GenBank accession number for Lys68 nucleotide sequence, KJ475444).

*E. coli* BL21(DE3) harboring the endolysin vector was grown in 600 mL Luria Broth (LB) supplemented with 50 µg/mL kanamycin to an optical density (OD_600 nm_) of 0.6 (4 h, 120 rpm at 37°C). Recombinant protein expression was induced for 18 h at 16°C by the addition of isopropyl-β-D-thiogalactopyranoside (IPTG) to a final concentration of 0.5 mM. The culture was then centrifuged (9500×*g*, 30 min) and cells were disrupted by resuspending the pellet in 25 mL of lysis buffer (20 mM NaH_2_PO_4_, 0.5 M NaCl/NaOH, pH 7.4), followed by three cycles of freeze-thawing (−80°C to room temperature). Maintaining the sample on ice, cells were further disrupted by sonication (Cole-Parmer, Ultrasonic Processors) for 8–10 cycles (30 s pulse, 30 s pause). Insoluble cell debris were removed by centrifugation (9500×*g*, 30 min, 4°C). The supernatant was collected and filtered (0.22 µm filters), and applied to Ni^2+^-NTA resin stacked in 1 mL HisTrap HP columns (GE Healthcare, Waukesha, WI, USA) for purification, using protein-dependent imidazole concentrations in the washing and elution buffer (20 mM NaH_2_PO_4_, 0.5 M NaCl/NaOH, pH 7.4, 25–300 mM imidazole). Eluted protein was then dialysed against 10 mM PBS at pH 7.2 (using Maxi GeBAflex-tube Dialysis Kit - Gene Bio-Application L.T.D) and the protein concentration was determined using the BCA Protein Assay Kit with bovine serum albumin (BSA) as standard (Thermo Scientific).

### Site-directed mutagenesis

To establish the identification of the putative catalytic residues within the endolysin sequence, two active site mutations (Glu18Ala and Thr35Ala) were introduced. Two sets of overlapping mutagenic primers (5'-CGCGGCATTCGCGGGGTTCCGGG, forward, 5'-CCCGGAACCCCGCGAATGCCGCG, reverse, for Glu18Ala, and 5'-AGAATGAGAAGTACCTTGCTATTGGCTACGGCCAC, forward 5'-GTGGCCGTAGCCAATAGCAAGGTACTTCTCATTCT, reverse, for Thr35Ala, with mutation basepairs underlined), were applied using the QuickChange II XL Site-Directed Mutagenesis Kit (Agilent Technologies). Plasmids containing mutated endolysin genes were then introduced in competent *E. coli* BL21(DE3) cells for heterologous production as described above.

### Quantification and characterization of endolysin muralytic activity

The PG lytic (or muralytic) activity of the purified Lys68 was quantified using *P. aeruginosa* PAO1 cells permeabilized by chloroform/Tris treatments, resuspended in 80 mM phosphate buffer pH 7.2. This muralytic test has been extensively described for endolysins elsewhere [14]. Briefly, 30 µL of serial dilutions of enzyme were added to 270 µL of OM permeabilized cells. The activity of Lys68 was measured at room temperature through the decrease in OD_600 nm_ using a BIO-TEK Synergy HT Microplate Reader. The muralytic activity is based on the linear relation between lysis and concentration, according to a standardized calculation method described elsewhere [14]. Enzymatic activity is expressed as follows: activity (units/µM)  =  ((slope (OD_600 nm_/min)/µM))/0.001), where the activity of 1 unit is defined as the concentration of enzyme (in µM) necessary to create a drop in OD_600 nm_ of 0.001 per minute. Obtained values for the negative control (30 µL of PBS pH 7.2) were subtracted from the sample values.

The pH dependence of the muralytic activity was assessed using the same 30 µL of serial dilutions of enzyme on 270 µL of OM permeabilized cells, but now resuspended in a universal pH buffer (10 mM KH_2_PO_4_, 10 mM Na-citrate and 10 mM H_3_BO_4_) adjusted to different pHs within the range of 3 and 10.

Thermostability was evaluated by incubating Lys68 first at several temperatures (40°C, 60°C, 80°C and 100°C) for 30 min and later at 100°C during different time intervals (5, 10, 20, 30 and 45 min) in a MJ Mini BIO-RAD Thermocycler. This was followed by a 20-min cooling step on ice, after which the activity was measured using 30 µL of 2 µM Lys68 on 270 µL of OM permeabilized cells resuspended in 80 mM phosphate buffer pH 7.2. The residual muralytic activity of each sample relative to the activity of unheated reference sample at time 0 ( = 100% activity) was determined.

The determination of the endolysin lytic spectrum was conducted using the different strains listed in **Table S1**. Gram-negative cultures were grown overnight and diluted 1∶100 the following day in fresh 5 mL LB and allowed to grow until reaching the mid-exponential phase (OD_600 nm_ of 0.6). Cells were then plated (100 µL) onto LB agar Petri dishes and allowed to grow for 8 h to form bacterial lawns. After, a step involving treatment with chloroform vapours was included to permeabilize the OM [15], prior to adding a 30-µL drop of 2 µM of Lys68. Lysis halos were visualized after 30 min incubation period.

### Endolysin secondary structure characterization by circular dichroism

Circular dichroism (CD) experiments in the far- and near-UV region were performed using a Jasco J-815 CD spectrometer equipped with a water-cooled Peltier unit. The spectra were recorded in a cell width of 0.1-mm path length (110.QS, Hellma) from 185 to 360 nm for all proteins with 1 nm steps, scan speed of 20 nm/min, high sensitivity and a 16 s response time. Three consecutive scans for each sample and its respective buffer baseline were obtained. The averaged baseline spectrum was subtracted from the averaged sample spectrum measured under the same conditions. Secondary structure estimates were derived from the spectra using the CDSSTR [16] and CONTINLL [17], [18] routine of the DICHROWEB [19], [20] server run on the Set 4 optimised for a wavelength of 190–240 nm. Thermal denaturation/renaturation of the proteins was measured by monitoring the change in ellipticity in a cell width of 0.1 cm path- length at 222 nm over the range of 20°C to 75°C, in increments of 1°C/min. The experimental denaturation/renaturation profiles were analysed by a nonlinear least squares fit assuming a two-state transition and used to calculate the melting temperature (*T_m_*). For CD measurements, a concentration of 8 µM of Lys68 was prepared in presence of an universal buffer (10 mM KH_2_PO_4_, 10 mM Na-citrate and 10 mM H_3_BO_4_) adjusted to different pH values within the range of 3 and 10 (for pH dependence tests) and in 80 mM phosphate buffer pH 7.2 (for thermostability tests). The same conditions were used for the analysis of the pH and temperature on the muralytic activity described above.

### Endolysin antibacterial activity assay with OMPs

Gram-negative cells (listed in **Table S1**), grown overnight at 37°C, were diluted 1∶100 in fresh LB and allowed to grow to an OD_600 nm_ of 0.6. Cells were then resuspended in 10 mM HEPES/NaOH (pH 7.2) to a final density of 10^8^ colony forming units (CFU)/mL. Each culture (50 µL) was incubated for 30 min at room temperature with 25 µL of Lys68 (2 µM final concentration) together with 25 µL of water or 25 µL of OMPs. The final concentrations used were 0.5 mM EDTA, 2 mM of citric acid and 5 mM of malic acid (all prepared in water), concentrations described in literature as potential permeabilizers. Under the same conditions, the HEWL with similar catalytic activity (glycoside hydrolase) was assessed at a final concentration of 2 µM to compare with the Lys68 results.

Additionally and following the same protocol, the influence of different bacterial physiological states on the combinatorial effect of Lys68 and OMPs was evaluated after a 2 h incubation period against *Salmonella enterica* serovar Typhimurium LT2 cells grown for 3 h (planktonic exponential phase), 12 h (stationary phase) and 24 h (biofilm cells - in this case, 200 µL volume used in 96-microplate wells). In case of biofilm assays, 2 µL of overnight normalized cultures (OD adjusted to 1.0 in LB medium) were transferred to 96-well polystyrene plates (Orange Scientific) containing 198 µL of LB. To establish mature biofilms the plate was incubated for 24 h at 37°C and 120 rpms. Afterwards, the culture medium was removed and non-adherent cells removed by washing the biofilms twice with 200 µL of 10 mM HEPES/NaOH (pH 7.2). Cells were then incubated with 100 µL of HEPES together with 50 µL Lys68 (2 µM final concentration) and 50 µL of citric and malic acid (2 mM and 5 mM final concentrations, respectively).

For all experiments, water (instead of EDTA/citric/malic acid) or PBS (instead of Lys68 or HEWL) was used as a negative control. The effect of the Lys68 and outer membrane permeabilizer mixtures on Gram-negative cells (planktonic and biofilms) was assessed by serial dilution plating in 10 mM PBS pH 7.2 buffer to quantify the number of CFUs. The antibacterial activity was expressed as the relative inactivation in logarithmic units ( =  log_10_ (N_0_/N_i_) with N_0_  =  number of untreated cells (in the negative control) and N_i_  =  number of treated cells counted after incubation). Averages ± standard deviations for all experiments are given for n = 4 repeats.

### OM permeabilization disruption mechanism

In order to assess the citric and malic acid permeabilization mechanism, three independent assays with *Salmonella* Typhimurium LT2 cells were made, as previously described with some modifications. Firstly, the reaction was supplemented with 5 mM of MgCl_2_, an extra source of divalent cations. Secondly, the cells were incubated with 3.5 and 4 mM HCl instead of the OMP agents, to mimic the pH drop achieved by 2 mM citric acid (pH 4.2) and 5 mM malic acid (pH 3.8), respectively. All reactions followed an incubation period of 30 min, after which cell suspensions were diluted, plated on LB agar plates and the CFUs were counted as previously described.

Finally, a third complementary reaction was carried out, to visualize the OMPs effect. Here, *S.* Typhimurium LT2 cells were incubated with 2 mM citric acid and 2 µM KZ-EGFP protein (instead of Lys68). KZ-EGFP is a previously described PG binding domain from a *Pseudomonas* phage endolysin KZ144 coupled to a green fluorescent protein that has strong affinity for Gram-negative PG [21]. A negative wild-type EGFP control was also included for comparison. After a 30-min incubation period, the sample was washed twice in 10 mM HEPES/NaOH (pH 7.2). Then, a 30-µl drop was spotted onto a microscope slide and images were recorded using confocal laser scanning microscopy (FluoView FV1000 microscope, Olympus) in search for fluorescent *Salmonella*. *Salmonella* cells treated with water (instead of citric acid) and EFGP were used as a negative control.

## Results

### *In silico* analysis

The 489-bps endolysin gene (*lys68*) from *Salmonella* phage phi68, encoding a 162-amino acid protein with a deduced molecular mass of 19.6 kDa, was amplified and sequenced. Afterwards, a bioinformatic analysis was conducted to analyse the primary and secondary structures of Lys68 as well as a possible identification of its putative catalytic residues.

Primary structure comparisons using BlastP, revealed high homology (>90%) to six other endolysin proteins from *Salmonella* phages, namely SETP3, ST4, SE2, vB_SenS-Ent1, SS3e and wksl3, that have not yet been characterized *in vitro*. Lys68 is predicted to be a globular protein with a conserved domain between amino acids 1–151 and belongs to the glycoside hydrolase family GH24 (**Figure S1**) [22]. This class of enzymes degrades the cell wall by catalyzing the hydrolysis of 1, 4-linkages between *N*-acetylmuramic acid and *N*-acetyl-D-glucosamine residues.

Secondary structure analysis using HHpred output with PDB as a database, which contains all publicly available protein structures, identified endolysins from *Enterobacteria* phages P1, P22 and T4 as relevant homologs that have high α-helical content. This is in agreement with the 9 α-helices (54.9%) and 7 β-sheets (4.3%) regions predicted using the PSIPRED server (**Figure S1a**).

T4, P1 and P22 endolysins are well-known muramidases (GH24) with different catalytic residues identified. While in T4 endolysin experimental evidence shows a catalytic triad (Glu-11-Asp-20-Thr-26) being responsible for glycosidase reaction [23], the P1 and P22 active sites have only been predicted by sequence similarity as a catalytic diad (Glu-42-Thr-57) and triad (Glu-35-Asp-44-Thr-50) respectively. In case of Lys68, BlastP analysis identified the residues Glu-18 and Thr-35 as highly conserved, and indicates them as probable catalytic residues of Lys68 (**Figure S1b**). No aspartic acids were found between the Glu and Thr residues.

### Lys68 spectra and pH dependence on the activity/stability

Overexpression of Lys68 in *E. coli* BL21(DE3) yielded a soluble protein of 14.3 mg/L with >95% purity, as judged by SDS-PAGE analysis (data not shown). To confirm the predicted catalytic activity, Lys68 endolysin 30 µL drops (2 µM) were spotted on several OM-permeabilized Gram-negative cells, and all were efficiently lysed.

**Figure 1:**
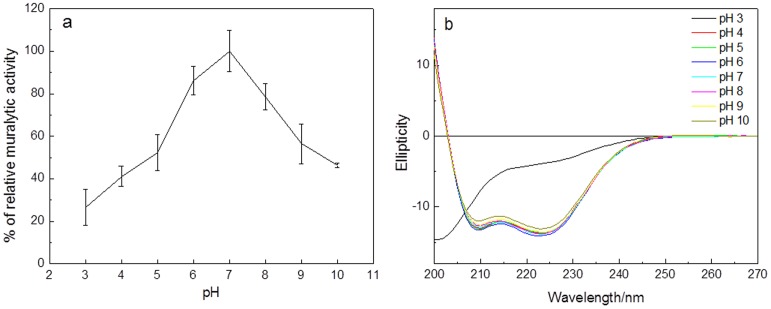
Influence of pH on the muralytic activity and secondary structure stability of Lys68. a) pH optimization curve for enzymatic activity of Lys68. Relative muralytic activity is measured as the slope of the OD_600nm_/min curve, given in percentage by comparing to the activity at pH 7.0 (the highest measured value) (Y-axis) on OM-permeabilized *P. aeruginosa* PAO1 substrate and is shown for a pH range between 3 and 10 (X-axis). Averages and standard deviations of three repeated experiments are given. b) CD spectra of Lys68 as a function of pH using a universal buffer with adjusted pH (3.0-10.0).

Regarding pH dependence of the muralytic activity, Lys68 remains active over a pH range from 4.0 to 10.0 (maintaining 41.17%±4.81 and 46.31%±0.96 of its activity, respectively) (**Figure 1a**), with a pH optimum around 7.0 (pH of the bacterial cytoplasm). The muralytic activity of Lys68 at optimal pH is 400 Units/µM and decreases by 23.3% (non-significant) and 74.9% (significant) when the protein is stored for two months at 4°C and −20°C, respectively (**Figure S2**). Therefore, even when preparing single-use aliquots of Lys68 at −20°C, in the absence of glycerol, protein functionality is significantly influenced by either an increase the protein concentration ionic strength or a pH shift after formation of ice crystals during storage.

To elucidate the major structural features of Lys68, we also carried out CD studies using a universal buffer at different pH values (**Figure 1b**). The Lys68 CD profile exhibited two negative dichroic minima at 222 nm and 208 nm and a positive dichroic maximum at 192 nm, which is characteristic of a protein with high α-helix content. Deconvolution of the CD spectra, using a dataset as specified in Materials and Methods, allowed us to determine that 37% of Lys68 folds as α-helices, 17% as β-sheet, 18% as turns, while 27% was unordered, which matches with the α-helix content predicted in the PSIPRED. Interestingly, when the pH was between 4.0 and 10.0, the obtained spectra were almost identical (insignificant ellipticity values changes of approximately 1%) and the predicted secondary structures content remained essentially unchanged. These data show that the secondary structure content of Lys68, at various pH conditions, is highly stable, even at pH 4.0. Conformational changes occurred around pH 3.0 with loss of secondary structure. The ellipticity at 222 nm at pH 3.0 decreased compared to values observed between pH 4.0–10.0.

### Thermostability

**Figure 2:**
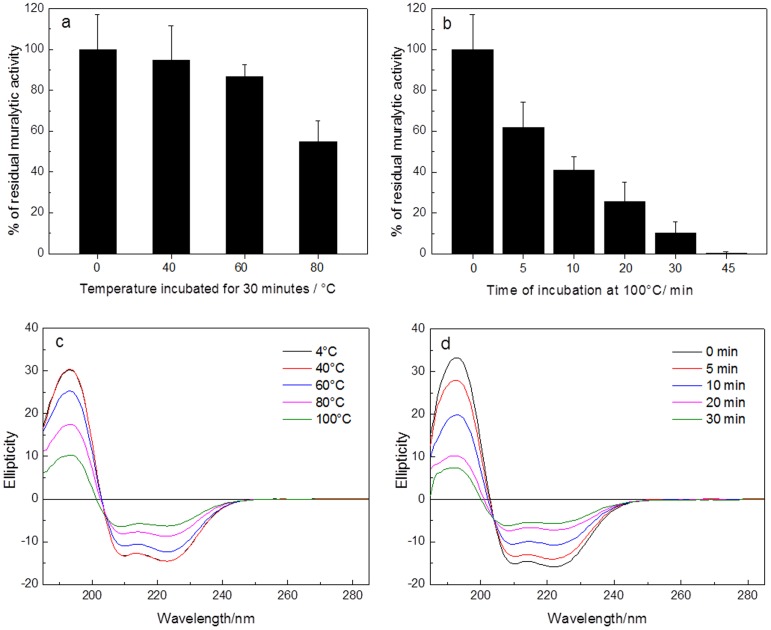
Influence of temperature on the muralytic activity and secondary structure stability of Lys68. a) The residual muralytic activity Lys68 (2 µM) on OM permeabilized *P. aeruginosa* PAO1 cell substrate after incubation at 4, 40, 60 and 80°C for 30 min and b) after heat treatment at 100°C for 0, 5, 10, 20, 30 and 45 min, followed by a 20-min cooling step on ice. Four repeated and independent experiments are shown; c, d) CD spectra of Lys68 incubated under the same conditions.

Thermostability tests were also evaluated on the endolysin muralytic activity and stability. Activity assays showed non-significant reductions on the enzymatic activity after 30 min incubation of the enzyme at 40°C and at 60°C, maintaining 54.7% of its residual activity at 80°C. When heated to 100°C, the muralytic activity significantly dropped to 25.8%, 10.2% and 0.27% of the initial activity after 20, 30 and 45 min incubation, respectively (**Figure 2a, 2b**).

CD spectra of Lys68 (protein ellipticity) was also measured under the same temperatures (40, 60, 80 and 100°C) (**Figure 2c, 2d**). It was observed that from 4°C to 40°C, Lys68 was very stable not changing its secondary structure. As the incubation temperature was increasing from 40°C to 60°C, 80°C and 100°C, the changes in secondary structures were evident with a continuous decrease in the ellipticity of the 208 nm and 220 nm bands. In accordance, the CD thermal stability shows that the loss of muralytic activity upon thermal stress is accompanied with gradual loss of endolysin secondary structure.

**Figure 3:**
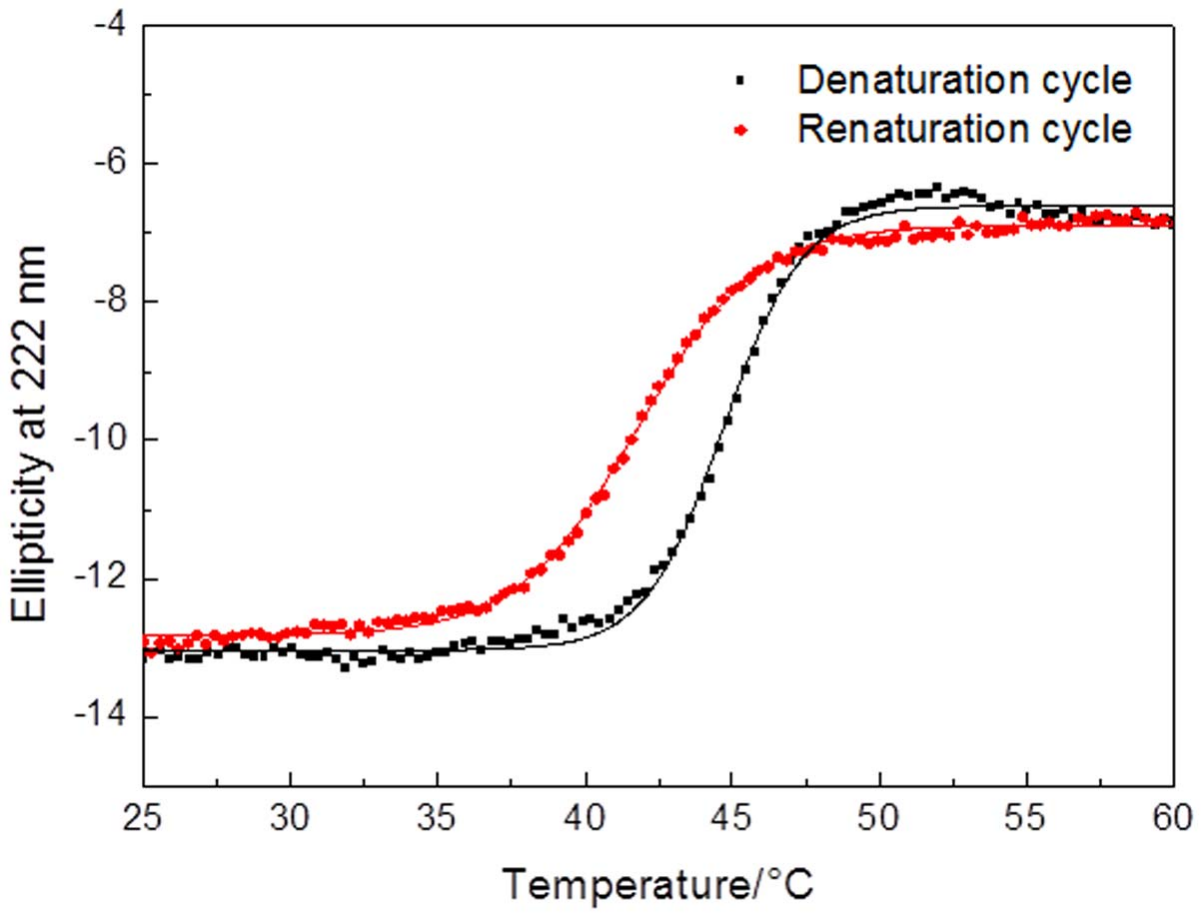
Thermal denaturation and renaturation profiles of Lys68. The melting curve for Lys68 is measured by monitoring the absorbance at 222 nm against increasing (denaturation) and decreasing temperatures (renaturation) at pH 7.0.

To confirm a possible refolding mechanism after thermal stress, the loss of Lys68 CD signal (ellipticity) at 222 nm (dichroic band characteristic for α-helical proteins) was monitored, when increasing the temperature of the sample from 20°C to 75°C at optimal pH for muralytic activity (pH 7.0) Lys68 demonstrated a sigmoidal thermal denaturation profile, reflecting protein unfolding (**Figure 3**). The *T_m_* was found to be around 44°C according to a two-state model. Interestingly, the renaturation experiments showed a maximum recovery of the native features after the protein was heated up to 75°C under the same experimental conditions, and a loss in ellipticity of only 1.3%. Therefore, a confirmation of protein refolding mechanism is given, explaining how the endolysin can retain activity after exposure of temperatures higher than its *T_m_* of 44°C.

### Combinatorial effect of OMPs with Lys68 and HEWL on Gram-negative bacteria

The *in vitro* antibacterial activity of OMP/Lys68 and OMP/HEWL was investigated on several Gram-negative bacteria genera (**Table 1**). In the absence of an OMP agent, the activity of Lys68 or HEWL against exponentially growing cells was insignificant, as expected, due to presence of a protective OM that prevents the endolysin from reaching the PG. In the presence of different OMPs (EDTA, citric and malic acid), a strong antibacterial activity was observed and dependent on the type of OMP used. EDTA/Lys68 had a more pronounced killing effect on *Pseudomonas* cells than EDTA/HEWL. Both citric and malic acid were significantly better than EDTA in enhancing the access of Lys68 to the PG, but this was not observed for HEWL. Citric acid/Lys68 was not only able to kill *Pseudomonas* cells, but also caused a significant reduction of *S.* Typhimurium LT2 (2.89±0.27 Log reduction) and *A. baumannii* (1.01±0.33 Log reduction) as well of *Shigella sonnei* (1.40±0.33 Log reduction), *E. coli* O157:H7 (1.18±0.12 Log reduction) and *Cronobacter sakazakii* (1.09±0.19 Log reduction). The effect of malic acid/Lys68 was higher in all strains compared to EDTA/Lys68 and citric acid/Lys68. Reductions of 2–3 logs units were observed on *P. aeruginosa* (3.31±0.21), *S.* Typhimurium LT2 (2.62±0.32) and *A. baumannii* (2.75±0.29). Overall, higher log reduction values were obtained with malic acid in combination with Lys68 after 30 min of incubation, killing 11 different Gram-negative bacterial species. In contrast to the other strains, none of the previous OMPs could sensitize *Klebsiella oxytoca* and *Yersinia enterocolitica* strains to Lys68.

**Table 1:** Combinatorial antibacterial activity of outer membrane permeabilizers with (A and B) or without (C) HEWL/Lys68 on Gram-negative bacterial pathogens.

A Bacterial Species	HEWL/Water	HEWL/EDTA	HEWL/Citric	HEWL/Malic
***Salmonella*** ** Typhimurium ** ***LT2***	0.03±0.07	0.14±0.23	0.08±0.15	0.07±0.09
***Acinetobacter baumannii 2***	0.01±0.09	0.04±0.06	0.10±0.06	0.17±0.15
***Pseudomonas aeruginosa*** ** PAO1**	0.02±0.10	**0.89±0.17**	0.24±0.02	**2.19±0.32**
***Pseudomonas fluorescens*** ** 7A**	0.04±0.04	0.30±0.38	0.28±0.13	**2.88±0.35**
***Shigella sonnei*** ** ATCC 25931**	0.22±0.29	0.01±0.11	0.02±0.07	0.06±0.12
***E. coli*** ** O157:H7 CECT 4782**	0.04±0.15	0.11±0.12	0.10±0.05	0.09±0.04
***Cronobacter sakazakii*** ** CECT 858**	0.11±0.18	0.15±0.20	0.30±0.16	0.20±0.13
***Pantoea agglomerans*** ** SA5634**	0.04±0.10	0.08±0.15	0.20±0.19	0.22±0.29
***Enterobacter amnigenus*** ** CECT 4878**	0.04±0.07	0.07±0.13	0.18±0.17	0.26±0.04
***Proteus mirabilis*** ** SA5445**	0.08±0.03	0.09±0.14	0.03±0.25	0.17±0.14
***Salmonella bongori*** ** SGSC 3100**	0.02±0.10	0.10±0.09	0.09±0.13	0.47±0.19
***Klebsiella oxytoca*** ** ATCC 13182**	0.01±0.02	0.10±0.05	0.13±0.08	0.26±0.06
***Yersinia enterocolitica*** ** SA5429**	0.06±0.05	0.18±0.11	0.11±0.12	0.34±0.11

B	Lys68/Water	Lys68/EDTA	Lys68/Citric	Lys68/Malic
***Salmonella*** ** Typhimurium ** ***LT2***	0.14±0.16	0.16±0.12	**2.89±0.27**	**2.62±0.32**
***Acinetobacter baumannii 2***	0.04±0.12	0.14±0.22	**1.01±0.33**	**2.75±0.29**
***Pseudomonas aeruginosa*** ** PAO1**	0.04±0.34	**2.41±0.26**	**1.48±0.35**	**3.31±0.21**
***Pseudomonas fluorescens*** ** 7A**	0.02±0.10	**1.55±0.37**	**1.40±0.23**	**5.44±0.24**
***Shigella sonnei*** ** ATCC 25931**	0.13±0.28	0.35±0.44	**1.40±0.33**	**2.35±0.31**
***E. coli*** ** O157:H7 CECT 4782**	0.02±0.02	0.21±0.14	**1.18±0.12**	**2.37±0.35**
***Cronobacter sakazakii*** ** CECT 858**	0.16±0.06	0.30±0.09	**1.09±0.19**	**1.40±0.30**
***Pantoea agglomerans*** ** SA5634**	0.03±0.18	0.13±0.12	0.32±0.18	**1.26±0.18**
***Enterobacter amnigenus*** ** CECT 4878**	0.04±0.11	0.28±0.18	0.68±0.36	**1.21±0.27**
***Proteus mirabilis*** ** SA5445**	0.03±0.12	0.09±0.04	0.41±0.29	**0.97±0.25**
***Salmonella bongori*** ** SGSC 3100**	0.06±0.10	0.08±0.16	0.13±0.09	**0.94±0.05**
***Klebsiella oxytoca*** ** ATCC 13182**	0.07±0.13	0.12±0.17	0.24±0.14	0.30±0.22
***Yersinia enterocolitica*** ** SA5429**	0.11±0.10	0.03±0.06	0.18±0.08	0.22±0.07

C	------	PBS/EDTA	PBS/Citric	PBS/Malic
***Salmonella*** ** Typhimurium ** ***LT2***		0.09±0.10	0.07±0.05	0.06±0.09
***Acinetobacter baumannii 2***		0.04±0.03	0.07±0.05	0.07±0.04
***Pseudomonas aeruginosa*** ** PAO1**		0.05±0.06	0.60±0.14	**1.83±0.31**
***Pseudomonas fluorescens*** ** 7A**		0.42±0.31	0.70±0.14	**2.70±0.31**
***Shigella sonnei*** ** ATCC 25931**		0.01±0.14	0.02±0.14	0.14±0.03
***E. coli*** ** O157:H7 CECT 4782**		0.06±0.04	0.01±0.09	0.18±0.18
***Cronobacter sakazakii*** ** CECT 858**		0.17±0.12	0.06±0.14	0.28±0.10
***Pantoea agglomerans*** ** SA5634**		0.07±0.10	0.03±0.18	0.10±0.18
***Enterobacter amnigenus*** ** CECT 4878**		0.08±0.08	0.15±0.15	0.16±0.18
***Proteus mirabilis*** ** SA5445**		0.02±0.10	0.01±0.23	0.03±0.12
***Salmonella bongori*** ** SGSC 3100**		0.07±0.12	0.05±0.14	0.16±0.03
***Klebsiella oxytoca*** ** ATCC 13182**		0.12±0.12	0.01±0.09	0.18±0.09
***Yersinia enterocolitica*** ** SA5429**		0.01±0.06	0.30±0.23	0.06±0.07

Exponential growing cells (10^8^ CFU/ml) were incubated with 2 µM HEWL/Lys68 in combination with one of the OMPs (0.5 mM EDTA, 2 mM citric acid or 5 mM malic acid). Protein incubation with water or OMPs with PBS served as negative controls. After incubation, the effect of HEWL/Lys68 and OMP mixtures was assessed by quantification of the number of CFUs. Antibacterial activity was quantified as the relative inactivation in logarithmic units ( =  log_10_(N_0_/N_i_) with N_0_  =  number of untreated cells and N_i_  =  number of cells after treatment). Averages ± standard deviations are given for n = 4 repeats. Indicated in bold are significant log reduction units observed (≥1 log).

Interestingly, extending the reaction period from 30 min to 2 h, the combinatory effect of organic acids and Lys68 against *S.* Typhimurium LT2 remarkably increased (**Table 2**). Maximum reductions of exponential cells were achieved when citric acid was combined with Lys68 (5.01±0.37 log reduction of viable cells) and this combination was also efficient against stationary cells (1.45±0.15 log units of viable cells) and biofilms (1.26±0.10 log units of viable cells).

**Table 2:** Influence of different *Salmonella* Typhimurium LT2 physiological states (planktonic/biofilm) on the combinatorial effect of Lys68 and outer membrane permeabilizers.

*S.* Tyhpimurium LT2	Planktonic		Biofilm
	Exponential phase	Stationary phase	
**Lys68 + Water**	0.11±0.14	0.14±0.07	0.15±0.11
**PBS + Citric acid**	0.10±0.11	0.08±0.11	0.09±0.19
**PBS + Malic acid**	0.08±0.20	0.19±0.18	0.15±0.25
**Lys68 + Citric acid**	**5.01±0.37**	**1.45±0.15**	**1.26±0.10**
**Lys68 + Malic acid**	**3.23±0.33**	0.65±0.28	**1.01±0.13**

In the planktonic and stationary assay, 50 µL cells resuspended in 10 mM HEPES/NaOH (pH 7.2) to a final 10^8^ CFU/ml were incubated with 25 µL Lys68 (2 µM final concentration) together with 25 µL of citric and malic acid (2 mM and 5 mM final concentrations, respectively) for 2 h. In biofilms assay, cells were washed twice with 200 µL of 10 mM HEPES/NaOH (pH 7.2). Cells were then incubated with 100 µL of Hepes together with 50 µL Lys68 (2 µM final concentration) and 50 µL of citric and malic acid (2 mM and 5 mM final concentrations, respectively) for 2 h. For both experiments, cells incubated with water (instead of citric/malic acid) or PBS (instead of Lys68) were used as negative controls. After incubation CFUs were counted. Averages ± standard deviations are given for n = 4 repeats. Indicated in bold are significant log reduction units observed (≥1 log).

### OM permeabilization mechanism

To further investigate the permeabilizing effect of citric and malic acid, *S.* Typhimurium LT2 cells were incubated with Lys68 in the presence of HCl (to drop the pH to 4.2 and 3.8, similar to citric and malic acid, respectively) for a 30 min period. The results summarized in **Table S2** show that the log reduction units of viable cells are mostly due to the acidity. In the presence of HCl, Lys68 is able to kill 2.55±0.37 log of viable cells at pH 4.2 (3.01±0.15 for citric acid) and a 2.31±0.22 log reduction of viable cells was obtained at pH 3.8 (2.60±0.17 for malic acid). In the case of HEWL, no activity towards *S*. Typhimurium LT2 was found in the presence of both organic acids and HCl.

To assess the effect of organic acids in permeabilizing Gram-negative OMs, an experiment was conducted incubating *S.* Typhimurium LT2 cells with citric acid in the presence of MgCl_2_. MgCl_2_ can contribute to the stability of the OM by providing electrostatic interactions of divalent cations (Mg^2+^) with proteins and the lipopolysaccharide (LPS) layer, and therefore, decrease the permeability of the OM to external agents. Consequently, the presence of 5 mM of MgCl_2_ abolished Lys68 antibacterial activity in all tested conditions (**Table S2**).

To visualise the effect of organic acids in permeabilizing Gram-negative OMs, an experiment was conducted incubating *S.* Typhimurium LT2 cells with citric acid in the presence of a fluorescent probe. This fluorescent probe was constructed earlier and consists of a *Pseudomonas* phage KZ144 endolysin cell wall (peptidoglycan) binding domain fused with an enhanced green fluorescent protein (KZ-EGPF) that has high-affinity to Gram-negative PG [21]. When incubating the cells for a 30-min period, green fluorescent *Salmonella* cells could be observed by epifluorescence microscopy (**Figure S3**), indicative of cell permeabilization.

### Lys68 PG digestion mechanism

To assess the Lys68 PG digestion mechanism and explain the activity of the endolysin at low pH, an analysis of its essential catalytic residues was made. Lys68 is classified as a member of the GH24 family [22], which has a glutamine (Glu) residue as the proton donor. Besides Glu, two other catalytic residues (Asp and Thr) may be involved in the catalytic reaction, as previously demonstrated with T4 lysozyme [23]. To identify a putative nucleophile for Lys68, BlastP searches were conducted. Results showed that, besides Glu-18, a second amino acid residue, Thr-35, was indicated as a putative catalytic residue of Lys68, with no Asp identified between both residues. Therefore, mutagenesis of both presumably essential catalytic residues was performed and resulted in one inactive enzyme (Glu18Ala) and one enzyme with similar activity (Thr35Ala) compared to the Lys68 wild-type (**Figure S4a**). Analysis of mutated proteins by CD did not provide any evidence of significant structural alterations due to the amino acid replacements (**Figure S4b**). Therefore the absence of activity was entirely attributed to the Glu18Ala substitution.

**Figure 4:**
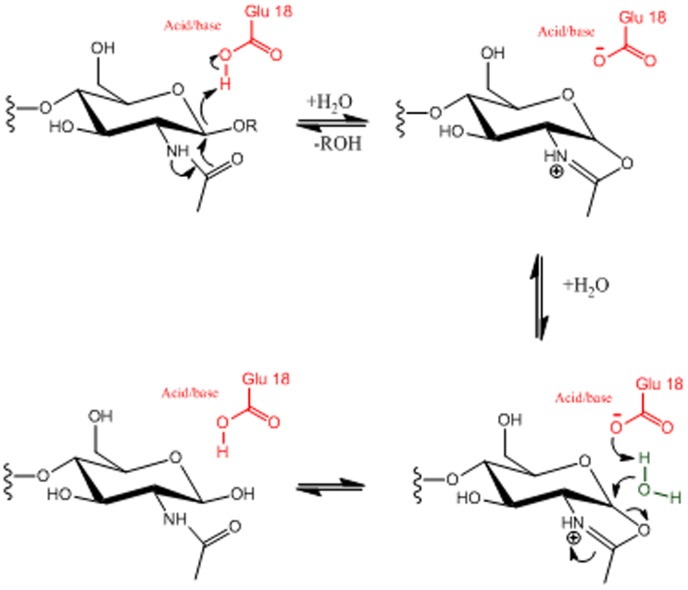
Proposed peptidoglycan hydrolases mechanism of Lys68. Lys68 hydrolyses the 1, 4-linkages between *N*-acetylmuramic acid and *N*-acetyl-D-glucosamine, through a two-step mechanism involving Glu-18 catalytic residue to cleave the peptidoglycan.

Based on the putative catalytic residues identified on other phage endolysins (muramidases) by protein similarity, our BlastP results that identified two catalytic candidates on Lys68 and the experimental data on its deleted mutants , we were not able to identify a second catalytic residue. Recently, Wohlkönig and coworkers, described the existence of two subgroups within the GH24 family; one that has a Glu-Asp-Thr catalytic triad and another one that only has Glu as a catalytic residue [24]. Therefore, we propose that Lys68 operates through a two-step mechanism involving Glu-18 (**Figure 4**). In the first step *N*-acetylhexosaminidases, which have an acetamido group, are capable of neighboring group participation to form an intermediate oxazolinium ion, with acidic assistance provided by the Glu-18 carboxylate. In the second step the now deprotonated acidic carboxylate acts as a base and assists a nucleophilic water to hydrolyze the oxazolinium ion intermediate, giving the hydrolyzed product (through individual inversions that lead to a net retention of configuration).

## Discussion

Endolysins of phages infecting Gram-positive bacteria have been extensively studied [4]. In case of Gram-negative phage endolysins, although their enzymatic mechanisms have been intensively studied, they efficacy in terms of antibacterial (biocontrol) potential have been scarcely explored. This has to do withthe impermeable bacterial OM protects the underlying PG layer from enzymatic hydrolysis by the exogenous endolysin. In this work, a new endolysin from a Gram-negative infecting phage is described. *In silico* characterization of Lys68 from the *Salmonella* phage phi68 shows elevated amino acid sequence similarity (>90% identity) to other *Salmonella* phage endolysins belonging to the same lysozyme-like superfamily. The observed globular structure (catalytic domain alone) is also shared by most other Gram-negative phage endolysins, contrary to modular archetypes (catalytic and a binding domain) typically seen in endolysins from a Gram-positive background [4].

A biochemical characterization showed that Lys68 displays some interesting characteristics. Regarding substrate specificity, Lys68 degrades the PG of all 20 Gram-negative organisms tested, and this broad spectrum is explained by the common PG A1γ chemotype shared by these organisms [25]. The observed muralytic activity of the globular Lys68 (400 Units/µM) is lower compared to other described modular Gram-negative-like endolysins (e.g. KZ144 and PVPSE1gp146 with 2058 and 13613 U/µM, respectively, calculated using the same method) [8]. Although KZ144 and PVPSE1gp146 have different catalytic activities, belonging to the lytic transglycosylases and glycoside hydrolase family 19 respectively, a more obvious explanation for their higher activity is presence of cell wall binding domains. It has been previously demonstrated that modular Gram-negative endolysins possessing an N-terminal binding domain exponentially increases the muralytic activity [26]. These binding domains have been found in KZ144 and PVPSE1gp146, where in Lys68 is apparently absent. Nevertheless, when compared to the globular homologue HEWL (35 Units/µM, reported elsewhere), Lys68 is 12 times more active [14]. In addition, Lys68 also showed to be highly stable, a feature required for antimicrobial enzymes. From pH values of 4.0 to 10.0, Lys68 does not change its secondary structure, where it has an optimal pH for muralytic activity around 7.0, without significant loss of its activity even when stored for two months at 4°C.

Once subjected to thermostability tests, in general, some endolysins maintain their activity after long heating periods. Within endolysins from a Gram-negative background, *Pseudomonas* phage endolysins KZ144 and EL188 activity still remains high after being exposed to 50°C for 10 min [26]. In contrast, the phage T4 lysozyme only retains a minor fraction of its activity after a 5 min treatment at 65°C [27]. Recently, a highly thermostable modular endolysin (PVP-SE1gp36) that withstands temperatures up to 100°C was described [8]. Lys68 is a globular protein where the muralytic activity is only completely inactivated after 30 min at 100°C. Because the melting temperature of the endolysin at pH 7.0 is 44°C, it seems that under the stress caused by higher temperatures, the endolysin is able to gradually regain its secondary structure to a point where, at high temperatures and prolonged exposures, it cannot refold. Recently, a similar folding/refolding mechanism was suggested by Schmelcher and coworkers http://www.plosone.org/article/info%3Adoi%2F10.1371%2Fjournal.pone.0036991 - pone.0036991-Schmelcher1to explain the high thermostability of the Gram-positive Listeria monocytogenes phage endolysins, HPL118 and HPL511 [28]. In their study, HPL118 and HPL511 retained 35% activity after 30 min at 90°C.

To allow a better diffusion of endolysins through the OM, a combinatorial approach was used on Gram-negative bacteria, using bacterial cell wall-degrading enzyme Lys68 or HEWL with an OMP. Both proteins combined with EDTA could reduce *P. aeruginosa* to some extent. A similar effect has previously been described with endolysin EL188 from *Pseudomonas* infecting phage EL of *P. aeruginosa* strain PAOI [7]. The positive effect of EDTA on the antibacterial activity is caused by the strong OM permeabilization capacity of EDTA through binding and withdrawal of the stabilizing divalent Mg^2+^ and Ca^2+^ cations present in the LPS layer of the Gram-negative OM [29]. As a result, the bacterial PG becomes more prone to the muralytic activity of the externally added endolysins. However, the sensitivity of other bacterial species to the destabilizing effect of EDTA was significantly lower, even when EDTA was increased to 5 mM (data not shown). It seems that the type and structure of the OM plays a key role for the antibacterial efficacy of the EDTA/enzyme combinatorial effect. Possibly, due to the low number of phosphate groups per LPS molecule and the corresponding amount of stabilizing divalent cations in the OM of *Enterobacteriaceae* compared to the *Pseudomonadaceae*, EDTA turns out to be ineffective to the former group. The observed synergetic effects of citric acid and malic acid, has never been described for phage endolysins. In this respect, both citric acid and malic acid had a better permeabilizing effect than EDTA, and were able to permeabilize several Gram-negative strains enhancing the activity of Lys68. Exceptions were *K. oxytoca* and *Y. enterocolitica* strains that may present some acid tolerance systems described (e.g. consuming or removing excess of protons by decarboxylation reactions and ion transporters, changing membrane composition), protecting them from the organic acids OM-destabilization [30].

In particular against exponential *S.* Typhimurium LT2 cells, an even more profound effect was observed when extending the reaction from 30 min to 2 h, although proving to be less efficient against stationary cells and biofilms. The lower efficiency in killing stationary cells can be a result of two aspects; *i)* structural changes in the OM (e.g. LPS biosynthesis), compromising the endolysin entry or LPS permeabilization through the organic acids, or *ii)* chemical modifications in the PG (e.g. glycosylated or acetylated glycans) known to contribute to high levels of resistance to glycosyl hydrolases [31], [32]. As for biofilms, besides the presence of several bacterial physiological states that can give rise to similar problems, additional diffusion limitations due to the high density matrix content can hinder the efficacy of endolysins and antimicrobials in general.

Although the permeabilizing capacity of citric acid by chelating divalent cations from the OM associated with its protonated form has been reported, additional acidity can contribute to OM damage [33]. It has been proposed that the LPS disintegration is accomplished by the ability of organic acids groups to migrate inside the cells to cause sublethal damages (e.g. enzyme inhibitions, amino acid decarboxylation, membrane disruption) [33], [34]. The 2 mM of citric acid (pKa 3.13 at 25°C) and 5 mM of malic acid (pKa 3.4 at 25°C) used, caused a drop of pH to 4.2 and 3.8, respectively. Because similar log reduction units were obtained using HCl instead of citric or malic acid combined with Lys68, the acidity effect (instead of its chelation effect) seems to play a key role in the OM permeabilization. Because millimolar concentrations of these organic acids by themselves did not have an antibacterial effect (with exception of *Pseudomonas*), they seem to be enough to compromise the cell wall to a level that allows Lys68 to act on the PG, causing bacterial death. Recently, a combined treatment with different organic acids to induce cell permeability in *E. coli* O157:H7 was reported [35]. Transmission electron microscopy images showed clear membrane disintegration that potentiated the bactericidal action of medium-chain fatty acids. When bacterial suspensions were supplemented with 5 mM of MgCl_2_, the effect of Lys68 combined with organic acids was completely abolished. MgCl_2_ has the ability to protect the bacterial OM damaged from the acid challenge, an effect already observed with lactic acid [34].

Based on the carbohydrate-active enzymes classification, several amino acids have been identified within lysozyme-like proteins to play a key role in the glycosidase reaction. HEWL (belonging to the GH22 class), which cleaves polysaccharide chains in bacterial cell walls, contains an active site that is constituted essentially by the two amino acids Glu-35 (pKa ∼6.2) and Asp-52 (pKa ∼3.6), that are only functional if the former is non-ionized and the latter is ionized [36]. Hence, the absence of HEWL enzymatic activity in the presence of malic and citric acid is related to the deionization of Asp52 at an acidic pH. Lys68 has a different catalytic system. Since the Thr-35 mutation did not affect Lys68 antimicrobial activities, we can conclude that Thr-35 is not involved in the catalysis of PG. Therefore, it is tempting to speculate that Lys68 operates through a two-step mechanism involving Glu-18, capable of digesting the 1, 4-linkages between *N*-acetylmuramic acid and *N*-acetyl-D-glucosamine residues of the PG in a low pH environment (pH around 4.0) through a retaining mechanism. There is a lack of studies conducted to confirm the phage endolysins (muramidases) catalytic sites. However, the catalytic activity of amino acid Glu-73 in the goose egg-white lysozyme has been demonstrated by Weaver and co-workers to be sufficient to digest PG bonds [37].

To summarize, Gram-negative bacterial pathogens prevail in various surroundings and their resilience is aided by the presence of an OM that prevents toxic substances from entering the cell. We show that an endolysin from a Gram-negative background can have a broad activity and good (thermo)stability properties. We further demonstrate that Lys68 can have an effective anti-Gram-negative activity when combined not only with EDTA, but specially with citric acid and malic acid, that can now be further explored to other endolysins for several potential applications. EDTA/Lys68 could be used as a therapeutic product to control *P. aeruginosa*, typically present in burn wound infections or as food preservative against *P. fluorescens*. The broad antimicrobial effect of citric acid/Lys68 and malic acid/Lys68 acid could be applied in food and clinical settings as an effective sanitizer to prevent food spoilage and nosocomial bacterial infections. Additionally, the powerful effect of Lys68 with organic acids can be used therapeutically to treat for example topical infections as chronic wounds, when applied in a cream or ointment. Altogether, this study demonstrated that bacterial cell wall hydrolase from phage origin (Lys68) combined with citric or malic acid have a powerful killing activity and can be used, as alternatives to chemical antimicrobial agents, to fight Gram-negative pathogens.

## Supporting Information

Figure S1 ***In silico*** **analysis of the Lys68.** **a**) Amino acid sequence of endolysin Lys68, where a phage-related lysozyme domain, belonging to the Glycoside Hydrolase Family 24 (GH24), was identified using HHpred webserver with the Pfam, InterProScan and COG databases and an E-value of 2.3×10^−47^ and 100% of query coverage (AA 1–151 underlined). Secondary structure analysis based on PSIPRED predicts 9 α-helices and 7 β-sheets. BlastP output indicates conservation in both presumed catalytic residues glutamic acid (Glu-18) and Threonine (Thr-35) for the glycosylase reaction (marked in bold). **b**) Sequence alignment of high Lys68 homologs identified in HHpred output using PBD as database: *Enterobacteria* phages T4 (P00720.2), P1 (Q37875.1) and P21 (P27359.1) with the respective accession numbers given. The N-terminal domains are aligned with the Glu-8aa-Asp-5aa-Thr identified in T4 (highlighted in red), illustrating other putative catalytic residues by homology (highlighted in violet).
(PNG)

Figure S2 **Saturation curves for Lys68 muralytic activity under optimal pH (7.2).** The activity in OD_600 nm_/min (Y-axis) for incremental amounts of Lys68 (0 months – squares; 2 months at 4°C – circles; 2 months at −20°C – triangles) is depicted. Muralytic activity was quantified using outer membrane-permeabilized *P. aeruginosa* PAO1 cells as a substrate, resuspended in 80 mM phosphate buffer pH 7.2. Lys68 activity reaches saturation at 2 to 3 µM. A linear regression of the demarcated linear region of the saturation curves gives an activity of 400, 310 (23.3% less) and 100 Units/µM (74.9% less) at 0 months and after 2 months at 4°C and −20°C, respectively. Averages and standard deviations of three repeated experiments are given.
(PNG)

Figure S3 **Epifluorescence microscopy of the** ***S.*** **Typhimurium LT2 cells.** **a**) Cells incubated with 2 mM citric acid and 2 µM of KZ-EGFP protein; **b**) Cells incubated with water and 2 µM of KZ-EGFP; and **c**) Cells incubated with 2 mM citric acid and PBS for 30 min. Cell pellets were then washed twice and visualized using epifluorescence microscopy with a 1500 magnification. EGFP proteins are visualized in green targeted to the bacterial cell wall.
(PNG)

Figure S4 **Comparison of muralytic activity and the secondary structure of Lys68 wild-type and its mutants.** **a**) Muralytic activity on *P. aeruginosa* OM permeabilized cells resuspended in 80 mM phosphate buffer pH 7.2, measured as optical density decrease. **b**) Circular dichroism spectra of Lys68 wild-type and the two mutants (Glu18Ala and Thr35Ala).
(PNG)

Table S1 **Lytic activity of Lys68 against various Gram-negative strains.** Mid-exponential Gram-negative growing cells were plated onto LB agar Petri dishes to form bacterial lawns, after which their OM was permeabilized by chloroform treatments. Then, a 30-µL drop of 2 µM of purified protein was added on top of the lawn and incubated for 30 min, followed by a visualization analysis to spot lysis halos and assess bacterial susceptibility.
(DOCX)

Table S2 ***In vitro*** **antibacterial activity of Lys68 and HEWL in combination with HCl, citric or malic acid against** ***S.*** **Typhimurium LT2 cells.** Cell cultures (initial cell density of 10^8^ cells/mL) were incubated for 30 min with 2 µM Lys68 or 2 µM of HEWL in presence of either 3.5 mM of HCl (pH 4.2), 4 mM of HCl (pH 3.8), 2 mM of citric acid (pH 4.2) or 5 mM of malic acid (pH 3.8). The use of water instead of acids served as negative control. The antibacterial activity was expressed as the relative inactivation in logarithmic units ( =  log_10_ (N_0_/N_i_) with N_0_  =  number of untreated cells (negative control) and N_i_  =  number of treated cells counted after incubation). Averages and standard deviations of four repeated and independent experiments are shown. Log reductions considered significant (≥1 log unit) are marked in bold.
(DOCX)
