# Correction: A Thermostable *Salmonella* Phage Endolysin, Lys68, with Broad Bactericidal Properties against Gram-Negative Pathogens in Presence of Weak Acids

**DOI:** 10.1371/journal.pone.0115267

**Published:** 2014-12-04

**Authors:** The PLOS ONE Staff

There are errors in the Author Contributions. The correct contributions are: Conceived and designed the experiments: HO VT SS RL MNP LK JA. Performed the experiments: HO VT MW. Analyzed the data: HO VT SS RL MNP LK JA. Contributed reagents/materials/analysis tools: RL MNP LK JA. Wrote the paper: HO VT.
