# Supplementary figures and images for: A Thermostable Salmonella Phage Endolysin, Lys68, with Broad Bactericidal Properties against Gram-Negative Pathogens in Presence of Weak Acids
Source: PLoS One. 2014 Oct 7;9(10):e108376. doi: 10.1371/journal.pone.0108376 (PMC4188523; doi:10.1371/journal.pone.0108376)

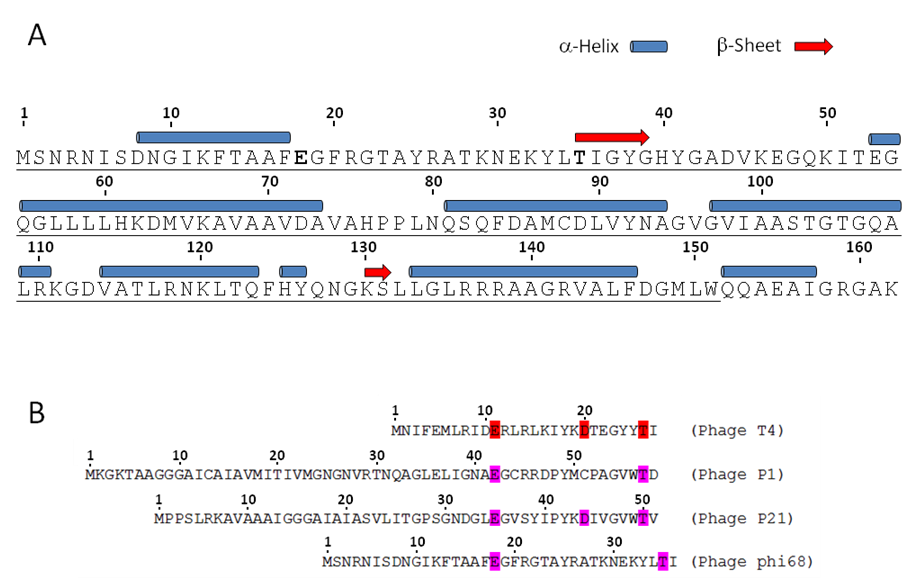

Supplement: Figure S1 — In silico analysis of the Lys68. a) Amino acid sequence of endolysin Lys68, where a phage-related lysozyme domain, belonging to the Glycoside Hydrolase Family 24 (GH24), was identified using HHpred webserver with the Pfam, InterProScan and COG databases and an E-value of 2.3×10^−47^ and 100% of query coverage (AA 1–151 underlined). Secondary structure analysis based on PSIPRED predicts 9 α-helices and 7 β-sheets. BlastP output indicates conservation in both presumed catalytic residues glutamic acid (Glu-18) and Threonine (Thr-35) for the glycosylase reaction (marked in bold). b) Sequence alignment of high Lys68 homologs identified in HHpred output using PBD as database: Enterobacteria phages T4 (P00720.2), P1 (Q37875.1) and P21 (P27359.1) with the respective accession numbers given. The N-terminal domains are aligned with the Glu-8aa-Asp-5aa-Thr identified in T4 (highlighted in red), illustrating other putative catalytic residues by homology (highlighted in violet). (PNG) [file pone.0108376.s001.png]

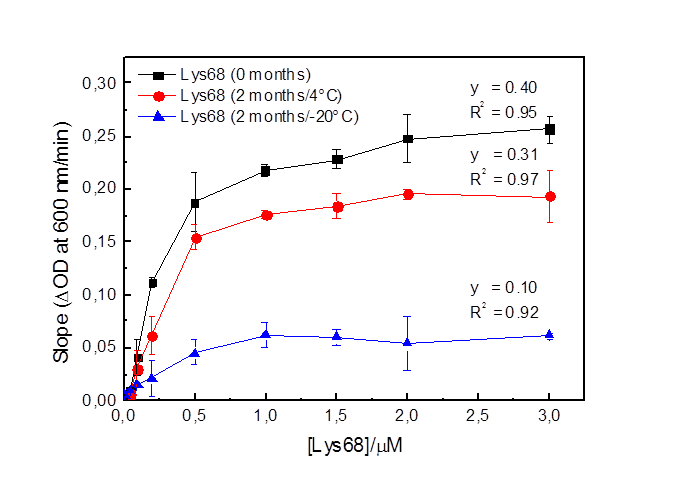

Supplement: Figure S2 — Saturation curves for Lys68 muralytic activity under optimal pH (7.2). The activity in OD600 nm/min (Y-axis) for incremental amounts of Lys68 (0 months – squares; 2 months at 4°C – circles; 2 months at −20°C – triangles) is depicted. Muralytic activity was quantified using outer membrane-permeabilized P. aeruginosa PAO1 cells as a substrate, resuspended in 80 mM phosphate buffer pH 7.2. Lys68 activity reaches saturation at 2 to 3 µM. A linear regression of the demarcated linear region of the saturation curves gives an activity of 400, 310 (23.3% less) and 100 Units/µM (74.9% less) at 0 months and after 2 months at 4°C and −20°C, respectively. Averages and standard deviations of three repeated experiments are given. (PNG) [file pone.0108376.s002.png]

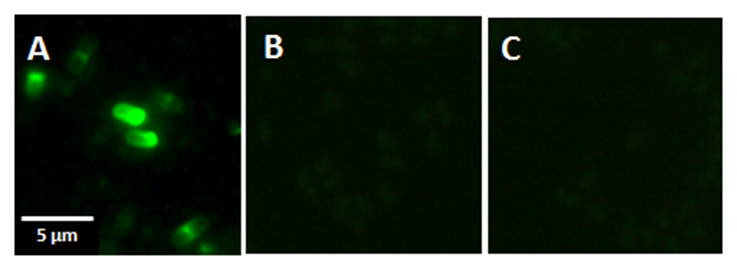

Supplement: Figure S3 — Epifluorescence microscopy of the S. Typhimurium LT2 cells. a) Cells incubated with 2 mM citric acid and 2 µM of KZ-EGFP protein; b) Cells incubated with water and 2 µM of KZ-EGFP; and c) Cells incubated with 2 mM citric acid and PBS for 30 min. Cell pellets were then washed twice and visualized using epifluorescence microscopy with a 1500 magnification. EGFP proteins are visualized in green targeted to the bacterial cell wall. (PNG) [file pone.0108376.s003.png]

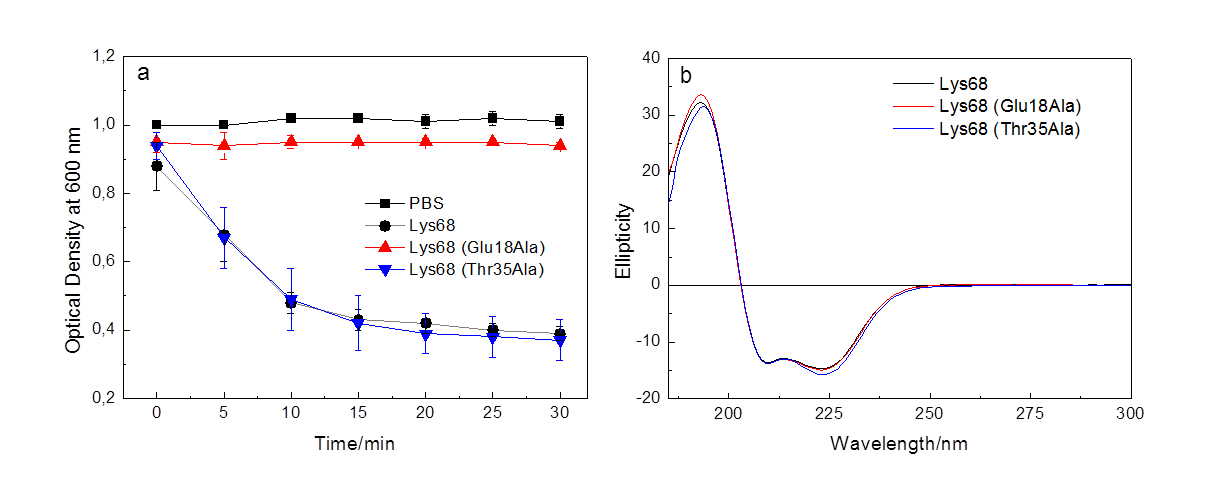

Supplement: Figure S4 — Comparison of muralytic activity and the secondary structure of Lys68 wild-type and its mutants. a) Muralytic activity on P. aeruginosa OM permeabilized cells resuspended in 80 mM phosphate buffer pH 7.2, measured as optical density decrease. b) Circular dichroism spectra of Lys68 wild-type and the two mutants (Glu18Ala and Thr35Ala). (PNG) [file pone.0108376.s004.png]
