# Supplementary material for: A Thermostable Salmonella Phage Endolysin, Lys68, with Broad Bactericidal Properties against Gram-Negative Pathogens in Presence of Weak Acids
Source: PLoS One. 2014 Oct 7;9(10):e108376. doi: 10.1371/journal.pone.0108376 (PMC4188523; doi:10.1371/journal.pone.0108376)
Supplement: Table S1 — Lytic activity of Lys68 against various Gram-negative strains. Mid-exponential Gram-negative growing cells were plated onto LB agar Petri dishes to form bacterial lawns, after which their OM was permeabilized by chloroform treatments. Then, a 30-µL drop of 2 µM of purified protein was added on top of the lawn and incubated for 30 min, followed by a visualization analysis to spot lysis halos and assess bacterial susceptibility. (DOCX) [file pone.0108376.s005.docx]

**Table S1. Lytic activity of Lys68 against various Gram-negative strains.**

| **Strain** |  | **Relative lysis activity** |
| --- | --- | --- |
| GRAM-NEGATIVE  *Escherichia coli*  *E. coli O157:H7*  *Salmonella* Typhimurium  *Salmonella* Enteritidis  *Salmonella bongori*  *Campylobacter jejuni*  *Cronobacter sakazakii*  *Cronobacter muytjensii*  *Pseudomonas fluorescens*  *Pseudomonas aeruginosa*  *Acinetobacter baumannii*  *Yersinia enterocolitica*  *Klebsiella oxytoca*  *Pantoea agglomerans*  *Enterobacter amnigenus*  *Proteus mirabilis*  *Citrobacter freundii*  *Shigella sonnei* | BL21(DE3)  CECT 4782^1^  LT2^1^  ATCC 13076  932*  SGSC 3100^1^  12662*  New M1*  CECT 858^1^  ATCC 51329  7A^1^*  PAO1^1^  2^1^*  SA5429^1^  ATCC 13182^1^  SA5634^1^  CECT 4878^1^  SA5445^1^  SA5345  ATCC 25931^1^ | S  S  I  S  S  S  S  S  S  S  S  S  S  S  S  I  S  I  S  S |

^1^strains tested with OMPs/enzymes; *strains from the Centre of Biological Engineering collection; S, susceptible (clear lysis halo); I, intermediate (turbid lysis halo); R, resistant (absence of lysis halo)
