# Supplementary material for: A Thermostable Salmonella Phage Endolysin, Lys68, with Broad Bactericidal Properties against Gram-Negative Pathogens in Presence of Weak Acids
Source: PLoS One. 2014 Oct 7;9(10):e108376. doi: 10.1371/journal.pone.0108376 (PMC4188523; doi:10.1371/journal.pone.0108376)
Supplement: Table S2 — In vitro antibacterial activity of Lys68 and HEWL in combination with HCl, citric or malic acid against S. Typhimurium LT2 cells. Cell cultures (initial cell density of 108 cells/mL) were incubated for 30 min with 2 µM Lys68 or 2 µM of HEWL in presence of either 3.5 mM of HCl (pH 4.2), 4 mM of HCl (pH 3.8), 2 mM of citric acid (pH 4.2) or 5 mM of malic acid (pH 3.8). The use of water instead of acids served as negative control. The antibacterial activity was expressed as the relative inactivation in logarithmic units ( = log10 (N0/Ni) with N0 = number of untreated cells (negative control) and Ni = number of treated cells counted after incubation). Averages and standard deviations of four repeated and independent experiments are shown. Log reductions considered significant (≥1 log unit) are marked in bold. (DOCX) [file pone.0108376.s006.docx]

**Table S2. *In vitro* antibacterial activity of Lys68 and HEWL in combination with HCl, citric or malic acid against *S.* Typhimurium LT2 cells**.

|  | **PBS** | **HEWL** | **Lys68** | **Lys68 + MgCl_2_** |
| --- | --- | --- | --- | --- |
| **HCl, pH 4.2** | 0.14 ± 0.18 | 0.12 ± 0.05 | **2.55 ± 0.37** | 0.10 ± 0.18 |
| **HCl, pH 3.8** | 0.01 ± 0.17 | 0.11 ± 0.10 | **2.31 ± 0.22** | 0.20 ± 0.13 |
| **Citric acid, pH 4.2** | 0.21 ± 0.14 | 0.09 ± 0.08 | **3.01 ± 0.15** | 0.24 ± 0.10 |
| **Malic acid, pH 3.8** | 0.11 ± 0.09 | 0.13 ± 0.06 | **2.60 ± 0.17** | 0.18 ± 0.16 |
